# Tuberculinum and Isopathy

**Published:** 1894-03

**Authors:** A. W. Holcombe

**Affiliations:** Kokomo, Ind.


### TUBERCULINUM AND ISOPATHY.

KOKOMO, IND., Jan. 11th, 1894.

EDITOR HOMŒOPATHIC PHYSICIAN:—The January number of the PHYSICIAN just received, and is a most interesting one. Dr. Holmes' article on Tuberculinum is very instructive. We need to know more about this wonderful remedy. I want to add my testimony to the good effects of Tuberculinum in ringworm. I had a patient, a little girl about ten years old, tall, very slender, black hair, long silken eyelashes, and large, brilliant brown eyes—a decided *brunette.* She had two large ringworms on the left fore-arm and was troubled with a dry cough—her father died of tuberculosis, and her sister was then in the last stage of the same disease. She received a powder of Tuberculinum$^{cm}$, dry, on the tongue. In about five days the ringworms entirely disappeared, as did the cough also; her general health improved, and she gained flesh rapidly. My experience has been that this remedy acts just as well with *brunettes* as with *blondes.*

I think the word *Isopathy,* as applied to the use of the nosodes in the high potencies, as Swan's DMM, for instance, is a *misnomer.* Through potentization the nature of the substance is changed, as Hahnemann says of Psorinum, so that in the sense of Isopathy we do not use an isopathic remedy at all. Dr. E. W. Sawyer has claimed for a number of years that the *very* high potency of a drug will *completely* antidote the crude effects of the drug, or, rather, the effects of the crude drug, and that it is the *only* thing that will. He and others have *demonstrated* this to be true, in actual practice, and that it is a

*law* of Similia. If this is true—and the results cannot be questioned—a change *must* have taken place through potentization. No one who has had any experience with the nosodes can doubt their efficacy, and they must act through some *law*. If they *cure*—and we know they *do*—it certainly is through the homœopathic law, as Hahnemann says there *is* no other law of *cure*. Why may not the nosode, changed by potentization, completely *antidote* the disease from which it was taken, as the *potency* does that of the crude drug? It is certainly in line of the homœopathic *law*, and as certainly, to my mind, *not* Isopathy.

Success to The Homœopathic Physician.

Sincerely,

A. W. Holcombe.

